# Rupture of a Huge Pancreatic Pseudocyst in a Superobese Patient: A Condition Mimicking Pulmonary Embolism

**DOI:** 10.7759/cureus.49643

**Published:** 2023-11-29

**Authors:** Vugar Suleimanov, Husain Naser, Ali Al-Taweel

**Affiliations:** 1 Surgery, Jubail General Hospital, Jubail, SAU; 2 General Surgery, Jubail General Hospital, Jubail, SAU

**Keywords:** rupture of pseudocyst, gall stone disease (gsd), diagnostic dilemma, pulmonary embolism (pe), giant pancreatic pseudocyst

## Abstract

Pancreatic pseudocysts are fluid-filled collections that can arise from acute or chronic pancreatitis and may lead to a range of complications, like rupture, infection, hemorrhage, etc. Morbid obesity may further complicate the diagnosis and management of such cases. The present report describes the case of a 26-year-old superobese female (BMI: 58 kg/m²) with a pancreatic pseudocyst that presented diagnostic challenges and mimicked pulmonary embolism when the pseudocyst had ruptured. The patient initially presented with persistent biliary colic due to gallstones. Despite undergoing laparoscopic cholecystectomy, she continued to experience symptoms, including nausea, bloating, and inability to tolerate food, and lab tests showed progressive elevation of serum bilirubin levels. A huge pancreatic pseudocyst was found to be obliterating the gastric cavity and compressing the common bile duct after the patient was subjected to further radiological imaging. While waiting to be transferred to a tertiary center with endoscopic retrograde cholangiopancreatography (ERCP), endoscopic stenting, and other facilities, she suddenly experienced severe symptoms, like shortness of breath, upper abdominal/chest pain, tachycardia (heart rate: 140 beats per min), dizziness, and low oxygen saturation. The likelihood of pulmonary embolism (PE) was very high in the differential diagnoses, but computer tomography pulmonary angiography (CTPA) ruled out PE. Based on imaging and clinical assessment, rupture of the pancreatic pseudocyst was diagnosed. The patient was subsequently managed in a tertiary hospital endoscopically. This case highlights the challenges of diagnosing and managing pancreatic pseudocysts in extremely obese patients. It also underscores the role of a multidisciplinary approach and vigilant clinical attention to prevent misdiagnosis and optimize outcomes.

## Introduction

Pancreatic pseudocysts represent a prevalent complication of acute and chronic pancreatitis, accounting for approximately 75% of all pancreatic masses. These pseudocysts are typically defined as a collection of pancreatic juice enclosed by a wall of fibrous or granulation tissue but lacking an epithelial lining [1]. According to Forsmark et al., acute pancreatitis is a common condition with varying degrees of severity, and its management often poses challenges related to diagnosis and treatment. While the case discussed represents a particularly complicated situation due to the patient's morbid obesity, pancreatic pseudocysts and pancreatitis at large are not uncommon in the general population [1]. They can present with a variety of symptoms ranging from abdominal pain and nausea to more severe complications such as gastric outlet or biliary obstruction, or hemorrhage [2].

Interestingly, pancreatic pseudocysts can lead to diagnostic challenges when presenting concomitantly with other medical conditions, such as gallstones. Gallstones alone are responsible for a significant number of hospital admissions due to gastrointestinal disease and are prevalent in morbidly obese patients [3]. The presence of a pancreatic pseudocyst can confound the diagnosis and management of gallstone disease, especially when obesity further complicates diagnostic imaging [4].

Morbid obesity is an escalating public health crisis with far-reaching implications for healthcare delivery. Patients with a body mass index (BMI) greater than 40 kg/m² are considered to be morbidly obese and are at elevated risk for several co-morbid conditions, including gallstone disease [5]. Morbid obesity poses significant challenges for diagnostic imaging, increasing the likelihood of false negatives or misinterpretations, as demonstrated in the present case [6]. Due to reduced penetration of ultrasound beam, ultrasonography in superobese individuals can lead to overlooked or misdiagnosed conditions, which have serious implications for patient care and outcomes [7].

Additionally, while complications like pulmonary embolism are commonly feared in the postoperative period for obese individuals, alternative diagnoses must be vigorously pursued when clinical presentation deviates from the expected [8]. Pulmonary embolism, a condition characterized by a sudden blockage in one of the pulmonary arteries, shares symptoms such as tachycardia and low oxygen saturation with other conditions, and its misdiagnosis can have catastrophic consequences [9].

This case explored a unique clinical scenario in which a 26-year-old superobese female, initially admitted for persistent biliary colic due to gallstones, was later found to have a giant pancreatic pseudocyst that mimicked symptoms of a pulmonary embolism postoperatively, when it ruptured. This case highlights the diagnostic challenges and therapeutic dilemmas in the management of complex patients with multiple comorbidities.

## Case presentation

A 26-year-old superobese female (with a BMI of 58 kg/m²) presented to the emergency room (ER) of a district general hospital with intermittent upper abdominal and right hypochondriac pain, nausea, and occasional vomiting after fatty meals for the last three months. The pain had increased in intensity and became continuous three days prior to coming to the ER. Her medical history was significant for gallstones diagnosed five months before, along with extreme obesity, for which she was on the waiting list for a bariatric procedure. She also had a prior hospital admission in other regions for acute cholecystitis two months ago, managed conservatively, and was advised to undergo laparoscopic cholecystectomy within six to eight weeks. Physical examination revealed a stable patient with normal heart rate, blood pressure, and core body temperature.

Initial abdominal ultrasonography demonstrated a distended gallbladder with normal wall thickness, multiple small gallstones, and a common bile duct (CBD) diameter of 5 mm. Lab tests showed near-normal liver function tests (LFT), normal complete blood count (CBC), and mildly elevated pancreatic enzymes (Table 1). She was scheduled for elective laparoscopic cholecystectomy (LC) in the next available slot, which was the next day. During the surgery, it was noted that the stomach was markedly distended, and the pylorus was obstructing access to the gallbladder neck area. Initially, we thought that the stomach and pylorus were distended due to Ambu bagging before intubation. Despite attempts to deflate the stomach via a nasogastric tube (NGT), the situation did not improve. The gallbladder was eventually removed with great difficulty due to limited access, but the procedure was performed safely, without any complications.

**Table 1:** Lab tests on admission and the day following LC. LC: laparoscopic cholecystectomy

Lab tests on admission	Result	Normal range	Units
White blood cells (WBC)	7.77	4-10	10^9^ cells/L
Hemoglobin (HGB)	12.60	12-16	g/dL
Platelets (PLT)	379	150-400	10^9^/L
Serum amylase (AMY)	170	28-100	U/L
Lipase	111.5	13-60	U/L
Random blood sugar (RBC)	5.71	3.9-7.8	mmol/L
Serum creatinine (Crea S)	48	62-106	µmol/L
Alkaline phosphatase (ALP)	84	40-129	U/L
Aspartate aminotransferase (AST)	23,10	0-41	U/L
Alanine aminotransferase (ALT)	15.20	0-41	U/L
Total bilirubin (TBIL)	12.10	0-21	µmol/L
Direct bilirubin (DBIL)	7.30	0-3.4	µmol/L
Gamma-glutamyl transferase (GGT)	92	8-61	U/L
Lab tests on day 1 post laparoscopic cholecystectomy			
Alkaline phosphatase (ALP)	193	40-129	U/L
Aspartate aminotransferase (AST)	146.70	0-41	U/L
Alanine aminotransferase (ALT)	66.70	0-41	U/L
Total bilirubin (TBIL)	47.50	0-21	µmol/L
Direct bilirubin (DBIL)	37.30	0-3.4	µmol/L
Gamma-glutamyl transferase (GGT)	285	8-61	U/L
White blood cells (WBC)	11.10	4-10	10^9^ cells/L
Platelets (PLT)	604	150-400	10^9^/L

**Figure 1:**
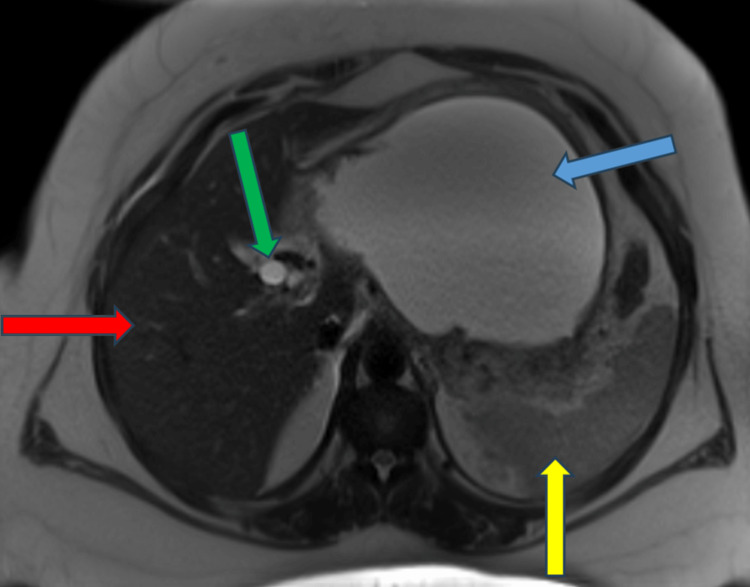
T2-weighted image on MRI scan shows a huge pancreatic pseudocyst (blue arrow); liver (red arrow), spleen (yellow arrow), and dilated CBD (green arrow) can be seen as well. CBD: common bile duct

Postoperatively, on day one, the patient was doing well and plan was to discharge her home. However, she developed nausea and bloating at the time of discharge, so we decided to keep her one day more and repeated her lab tests. Her liver function tests were found to be elevated, with total bilirubin being 47.5 µmol/L and direct bilirubin 37.30 µmol/L (Table 1). Magnetic resonance cholangiopancreatography (MRCP) was carried out, but the patient did not tolerate the procedure well. Only a few images could be taken, which revealed a large pancreatic pseudocyst compressing the stomach and duodenum, and a dilatation of CBD (Figure 1).

**Figure 2:**
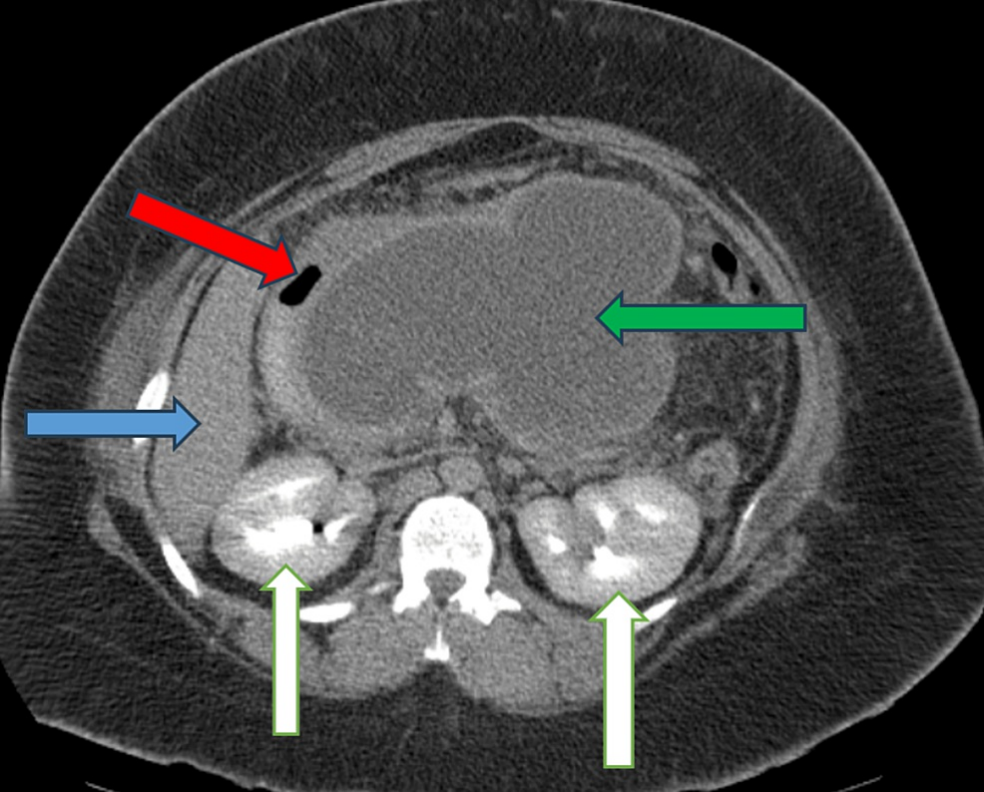
CECT shows a large pancreatic pseudocyst (green arrow) compressing the stomach (red arrow). Liver (blue arrow) and kidneys (white arrow) can be seen too. CECT: contrast-enhanced computer tomography

Since she did not tolerate lengthy MRCP scanning, we decided to perform a contrast-enhanced computer tomography (CECT) to delineate the pathology in detail. CECT showed a 19 x 15 x 12 cm pancreatic pseudocyst, which was significantly compressing the stomach and the distal CBD (Figure 2). Biliary sludge and small stones were also identified in distal CBD, which were thought to be contributing to the elevation of serum bilirubin levels. The pseudocyst had been missed in the initial ultrasound, mistaken for a fluid-filled stomach due to its large size and extreme obesity of the patient.

**Figure 3:**
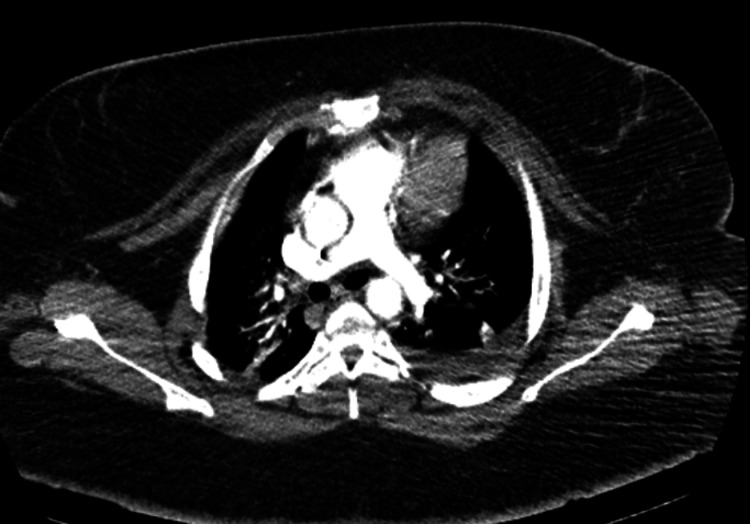
Normal CT pulmonary angiography.

**Figure 4:**
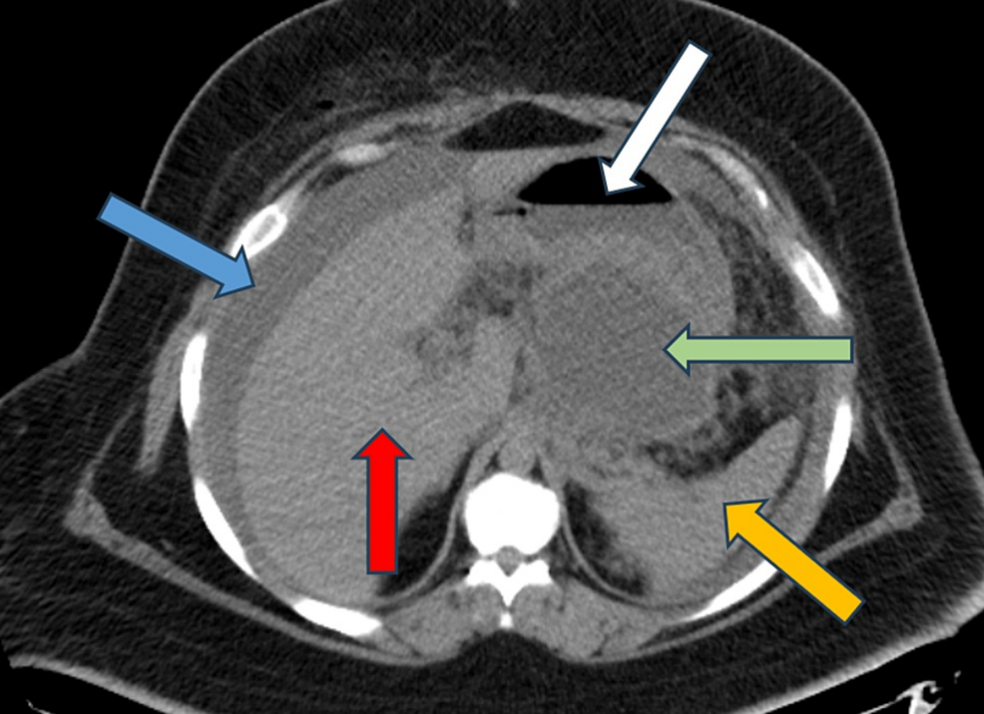
Axial cut of upper abdominal CT shows perihepatic free fluid (blue arrow), shrunken pseudocyst (green arrow), and stomach larger than before (white arrow). The spleen is depicted with an orange arrow, while liver is marked with a red arrow.

The patient's condition worsened steadily, and bilirubin levels continued to rise. She was not able to eat or drink. She needed intervention for her huge pancreatic pseudocyst, which was significantly compressing the stomach and duodenum, making oral feeding impossible, and CBD obstruction was causing steady elevation of serum bilirubin. Very tiny stones and biliary sludge in distal CBD could pass into the duodenum if there was no compression by a giant pseudocyst. Plans were made to relieve CBD obstruction initially by ERCP plus CBD drainage/stenting, then endoscopic drainage of the pseudocyst either at the time of ERCP or later after the settlement of the jaundice. Since we did not have a hepato-pancreato-biliary (HPB) surgeon or ERCP facilities in our hospital, arrangements were made to transfer the patient to a tertiary center. Before the transfer could take place, on day five after laparoscopic cholecystectomy, she developed severe upper abdominal and chest pain, shortness of breath, tachycardia with a heart rate reaching 140 beats per min, and low oxygen saturation. Given her postoperative and superobese status, pulmonary embolism was initially suspected (although she was on venous thromboembolism prophylaxis from the start, a fraction of very high-risk patients is well known to develop deep vein thrombosis {DVT} and PE despite prophylaxis). A computer tomography pulmonary angiography (CTPA) was carried out, which ruled out PE (Figure 3) but revealed free fluid in the abdominal cavity and a significant reduction in the size of the pancreatic pseudocyst, indicating its rupture (Figure 4).

The patient was managed in ICU with oxygen, analgesics, and IV fluids, and the transfer process was expedited. The patient remained stable with conservative management in the tertiary center. She was later successfully managed endoscopically, resolving her symptoms. On follow-up after three months in our hospital, she was symptom-free, waiting for her appointment with a bariatric surgeon. The timeline of events is given in the appendix.

## Discussion

Obese patients present a unique challenge to healthcare providers in terms of both diagnostic and therapeutic approaches to a variety of conditions, including bilio-pancreatic conditions. The challenge is exaggerated further in morbidly obese and superobese patients. Furthermore, rupture of a pancreatic pseudocyst can be easily mistaken for PE, which has a higher prevalence in postoperative cases and extreme obesity, missing such a complication can lead to a catastrophic consequence. The present case underscores several critical issues in the diagnostic and therapeutic management of gastrointestinal diseases, particularly when complicated by extreme obesity and coexisting conditions like pancreatic pseudocysts.

Diagnostic imaging, often the cornerstone of diagnosis in gastrointestinal diseases, presents unique challenges in morbidly obese patients. Ultrasonography, typically considered the first-line imaging modality for gallbladder disease, is known to be less accurate in obese patients [7]. In the present case, the initial ultrasonography failed to detect a large pancreatic pseudocyst, which was mistaken for a fluid-filled stomach. This aligns with previous literature highlighting the limitations of conventional imaging techniques in patients with high BMI [6].

Pancreatic pseudocysts can lead to a plethora of complications, including obstruction of the gastric outlet and the common bile duct (CBD), as seen in this case [1]. A significant challenge lies in the identification of such complications in the presence of other symptoms, like biliary colic, which could mask the underlying pseudocyst-induced pathology.

In a study by Cherata et al., a case was reported involving a patient with a pancreatic pseudocyst who presented with a right atrium floating thrombus and bilateral pulmonary embolism, mimicking a cardiac condition. This case highlighted the potential for pancreatic pseudocysts to mimic other serious medical conditions, emphasizing the importance of accurate diagnosis and appropriate management [10].

Madani et al. presented a case report of a pancreatic pseudocyst that mimicked a left kidney abscess. This case underscores the diagnostic challenges in distinguishing between pancreatic pseudocysts and other abdominal pathologies and the need for careful evaluation to prevent misdiagnosis and ensure appropriate treatment [11].

Graciano et al. reported a case of rupture of a pancreatic pseudocyst and discussed the therapeutic approach taken. This study sheds light on the clinical management of complications related to pancreatic pseudocysts and emphasizes the importance of timely intervention in such cases [12].

Chtourou et al. presented a case report of spontaneous rupture of an infected pseudocyst of the pancreas. The study highlights the potential for infection-related complications and underscores the need for vigilant monitoring and early intervention in patients with pancreatic pseudocysts, particularly in cases of infection [13].

A study by de Vries et al. in 1994 reported a case of pancreatic pseudocyst formation complicated by inferior vena cava thrombosis and pulmonary embolism. This early case report serves as a historical perspective on the recognition of pulmonary embolism as a potential complication of pancreatic pseudocysts, highlighting the importance of considering this diagnosis in patients with relevant symptoms [14].

Youness and Barrani reported a case of uncomplicated spontaneous rupture of a pancreatic pseudocyst into the duodenum. This case adds to the understanding of the diverse clinical presentations of pancreatic pseudocysts and the need for individualized management approaches [15].

The postoperative clinical deterioration of our patient was initially thought to be due to a pulmonary embolism, a diagnosis that is often considered in postoperative patients presenting with tachycardia and hypoxia [8]. However, this case demonstrates the necessity of considering an alternative condition, as the absence of pulmonary embolism on CT angiography led to the discovery of the ruptured pancreatic pseudocyst [9].

The effective management of complex cases such as this one necessitates a multidisciplinary approach involving gastroenterologists, radiologists, and surgeons specialized in hepato-pancreato-biliary surgery [2]. The initial lack of availability of ERCP and HPB specialists in our facility underscores the importance of ensuring comprehensive care settings for these challenging cases [3].

This case highlights the limitations of current diagnostic modalities in the setting of morbid obesity and coexisting gastrointestinal diseases. Future research should focus on developing more precise imaging techniques for this patient population. Additionally, healthcare providers should exercise a high index of clinical suspicion in complicated cases to avoid potential misdiagnosis [5].

## Conclusions

The lessons drawn from our case include the need for greater clinical suspicion in the diagnosis and management of pancreatic pseudocysts, especially in the setting of extreme obesity. If a patient with pancreatic pseudocyst develops sudden severe symptoms, the clinician must bear in mind the possibility of cyst rupture. This experience reiterates the critical role of a multidisciplinary approach involving surgeons, radiologists, and gastroenterologists for optimal patient care. Referral of such cases to a higher center with all the necessary facilities, like ERCP, endoscopic ultrasound, and hepato-pancreato-biliary service is recommended for a better outcome. Future studies should focus on improving diagnostic imaging techniques in obese populations and further elucidating the pathophysiology of pancreatic pseudocysts to advance treatment options.

## Appendices

**Table 2:** Timeline of patient's clinical events.

Timeline of patient’s clinical events		
December 31, 2022	Admission	Initial diagnosis: persistent biliary colic (diagnosed by ultrasonography)
January 01, 2023	Surgery	Laparoscopic cholecystectomy performed
January 02, 2023	Postoperative symptoms	Nausea, bloating, and elevated bilirubin levels
January 03, 2023	Revised diagnosis	Large pancreatic pseudocyst compressing CBD and stomach
January 05, 2023	Complication	Rupture of the pseudocyst
January 05, 2023	Transfer	Sent to tertiary hospital for specialized care

## References

[1] Forsmark CE, Vege SS, Wilcox CM (2016). Acute pancreatitis. N Engl J Med.

[2] Yadav D, Lowenfels AB (2013). The epidemiology of pancreatitis and pancreatic cancer. Gastroenterology.

[3] Everhart JE, Ruhl CE (2009). Burden of digestive diseases in the United States part iii: liver, biliary tract, and pancreas. Gastroenterology.

[4] Busireddy KK, AlObaidy M, Ramalho M, Kalubowila J, Baodong L, Santagostino I, Semelka RC (2014). Pancreatitis-imaging approach. World J Gastrointest Pathophysiol.

[5] Must A, Spadano J, Coakley EH, Field AE, Colditz G, Dietz WH (1999). The disease burden associated with overweight and obesity. JAMA.

[6] Kalb B, Martin DR, Sarmiento JM (2013). Paraduodenal pancreatitis: clinical performance of MR imaging in distinguishing from carcinoma. Radiology.

[7] Taylor SA, Carucci LR (2018). The role of imaging in obesity special feature. Br J Radiol.

[8] Tapson VF (2008). Acute pulmonary embolism. N Engl J Med.

[9] Konstantinides SV, Torbicki A, Agnelli G (2014). 2014 ESC guidelines on the diagnosis and management of acute pulmonary embolism. Eur Heart J.

[10] Cherata DA, Donoiu I, Mirea O, Diaconu R, Istratoaie O (2018). Right atrium floating thrombus and bilateral pulmonary embolism in a patient with pancreatic pseudocyst. J Cardiol Cases.

[11] Madani MA, Ouannes Y, Chaker K, Marrak M, Nouira Y (2023). Pancreatic pseudocyst mimicking a left kidney abscess: a case report. J Med Case Rep.

[12] Graciano VP, Rodante AC, Netto IF, Pin AS, Lacerda LO, Pontes GM, Marciano RD (2023). Rupture of pancreatic pseudocyst and therapeutic conduct: a case report. Braz J Health Biomed Sci.

[13] Chtourou MF, Beji H, Zribi S, Kallel Y, Bouassida M, Touinsi H (2023). Spontaneous rupture of an infected pseudocyst of the pancreas: a case report. Int J Surg Case Rep.

[14] De Vries JH, Ferwerda J, Rauws EA, Duijm LE (1994). Pancreatic pseudocyst formation complicated by inferior caval vein thrombosis and pulmonary embolism. Eur J Gastroenterol Hepatol.

[15] Youness AS, Barrani MM (2022). Uncomplicated spontaneous rupture of pancreatic pseudocyst into duodenum: a case report. Int J Adv Res.

